# PP17 Navigating Health Technology Assessment (HTA) Requirements During Development Through Early HTA Scientific Advice: Insights From Companies’ Strategies, Challenges, And Priorities

**DOI:** 10.1017/S0266462324001909

**Published:** 2025-01-07

**Authors:** Tina Wang, Neil McAuslane

## Abstract

**Introduction:**

Pharmaceutical companies have been actively taking early scientific advice from health technology assessment (HTA) agencies during development, focusing on study design to understand the HTA evidentiary requirements. The evolving advice landscape, including multistakeholder and international collaborations, highlights proactive engagement’s importance. This opinion survey assessed international pharmaceutical companies’ current experiences in seeking early HTA and explored strategies for forward-looking actions and considerations.

**Methods:**

An opinion survey was designed and conducted in 2023 as a cross-sectional questionnaire consisting of multiple-choice questions. The questionnaire provided a qualitative assessment of companies’ current strategies and experiences in taking early HTA advice as well as future considerations. Eligible survey participants were the senior management of Global HTA/market access departments at 22 top international pharmaceutical companies.

**Results:**

Responses were received from 13 companies. The National Institute for Health and Care Excellence (NICE) or the Federal Joint Committee (Gemeinsamer Bundesausschuss, G-BA) were utilized mostly for early HTA advice (92% respondents). Past European Medicines Agency HTA experience exists (50%), but there is no current engagement in Joint Scientific Consultation (JSC). However, JSC is deemed a top priority (83%) given the Regulation (EU) 2021/2282 on health technology assessment. Challenges in seeking advice include agency availability and internal resource and timing constraint. Advice-seeking actions mostly occurred during phase II trials but were frequently limited by agencies’ availability (84% respondents). Divergences were identified regarding eligibility criteria to seek advice and internal practices. Five success indicators were identified with the top-rated being the impact on development plan.

**Conclusions:**

This survey assessed companies’ practices in seeking early HTA advice, with most engaging national agencies like NICE and G-BA. The lack of current JSC participation, despite companies’ prioritization, highlights the agencies’ need to enhance capacity and resources. Survey results underscored the importance of companies’ adaptive strategies in the evolving environment, which can be supported by active measures of advice success.

